# Outcomes of Endovascular Treatment versus Standard Medical Treatment for Acute Ischemic Stroke with Basilar Artery Occlusion: A Systematic Review and Meta-Analysis

**DOI:** 10.3390/jcm12206444

**Published:** 2023-10-10

**Authors:** Jia-Hung Chen, Sheng-Chieh Lin, Chien-Tai Hong, Lung Chan

**Affiliations:** 1 Department of Neurology, Shuang-Ho Hospital, Taipei Medical University, New Taipei City 23561, Taiwan; 19587@s.tmu.edu.tw (J.-H.C.); b101102100@tmu.edu.tw (S.-C.L.); 15004@s.tmu.edu.tw (C.-T.H.); 2 Department of Neurology, School of Medicine, College of Medicine, Taipei Medical University, Taipei 11031, Taiwan

**Keywords:** endovascular treatment, standard medical treatment, basilar artery occlusion, functional outcome

## Abstract

Background: Although endovascular treatment (EVT) is beneficial for large vessel occlusion in anterior circulation stroke, whether these benefits exist for basilar artery occlusion (BAO) remains unclear. This systematic review and meta-analysis compared the outcomes of patients with BAO undergoing EVT and standard medical treatment (SMT). Methods: The PubMed, Embase, and Cochrane Library databases were searched for eligible randomized control trials (RCTs) and non-RCTs involving patients with acute ischemic stroke and BAO undergoing EVT or SMT. The following outcomes were assessed: 90-day functional outcomes (favorable outcome and functional independence: modified Rankin scale [mRS] score of 0–3 or 0–2, respectively), mortality, and symptomatic intracranial hemorrhage (sICH) incidence. The summary effect sizes were determined as risk ratios (RRs) through the Mantel–Haenszel method with a random-effects model. Results: Four RCTs and four non-RCTs were included. Compared with SMT, EVT resulted in a higher proportion of patients with 90-day mRS scores of 0–3 (RR: 1.54 [1.16–2.06] in RCTs and 1.88 [1.11–3.19] in non-RCTs), a higher proportion of patients achieving functional independence (90-day mRS score of 0–2; RR: 1.83 [1.07–3.12] and 1.84 [0.97–3.48], respectively), a lower risk of mortality (RR: 0.76 [0.65–0.89] and 0.72 [0.62–0.83], respectively), and a higher sICH risk (RR: 5.98 [2.11–16.97] and 4.95 [2.40–10.23], respectively). Severe neurological deficits, intravenous thrombolysis, and higher posterior circulation Acute Stroke Prognosis Early Computed Tomography Score (pc-ASPECTS) were associated with EVT benefits. Conclusion: In patients with BAO, EVT results in superior functional outcomes, lower mortality risk, and higher sICH risk than does SMT, independent of age and sex. Higher National Institutes of Health Stroke Scale scores, intravenous thrombolysis, and higher pc-ASPECTSs before treatment are associated with greater benefits from EVT.

## 1. Introduction

Basilar artery occlusion (BAO) is a serious cause of stroke; 1% of ischemic stroke cases and 5% of large vessel occlusion cases are caused by BAO [1,2]. Despite appropriate treatment, BAO continues to be associated with high rates of disability and mortality. The mortality rate during the initial month is high, reaching 35%, and it is equally troubling that nearly half of the patients who survive this critical period continue to experience functional dependency even after three months of treatment [3,4]. Although previous randomized control trials (RCTs) have demonstrated the greater efficacy of endovascular treatment (EVT) than of standard medical treatment (SMT) for large vessel occlusion in anterior circulation stroke within a specific time window [5,6,7,8,9], the benefit of EVT for posterior circulation stroke remains uncertain.

Several observational studies and RCTs have evaluated the efficacy and safety of EVT in patients with BAO. However, the results have been conflicting, and many of these studies had obvious limitations. In a 2009 observational registry study, intra-arterial treatment did not result in better 1-month functional outcomes than did medical therapy alone [10]. This finding was challenged by two extensive observational registry studies demonstrating that patients with BAO who underwent EVT had a higher probability of favorable outcomes and lower mortality rates [11,12]. Similarly inconsistent results have been observed in RCTs: two RCTs reported no differences in the outcomes of EVT and SMT for BAO, whereas a paired RCT reported superior outcomes of EVT [13,14,15,16]. 

Because the findings of various observational studies and RCTs regarding the effectiveness of EVT and SMT for patients with stroke and BAO are conflicting, further investigation is needed. Accordingly, in this systematic review and meta-analysis, we assessed the available evidence from RCTs and non-RCTs to provide valuable clinical guidance for treating BAO and aid health-care professionals in making informed decisions about the most effective therapeutic approach.

## 2. Materials and Methods

This systematic review and meta-analysis was performed in accordance with the Preferred Reporting Items for Systematic Reviews and Meta-Analyses guidelines. The review protocol was registered with PROSPERO (CRD42022343964).

### 2.1. Search Strategy and Study Selection

Two independent reviewers (J.-H.C. and S.-H.L.) searched the PubMed, Embase, and Cochrane Library databases for studies published before 1 May 2023, with no language restrictions. The search string was as follows: “(basilar OR vertebral OR vertebrobasilar) AND (stroke OR infarct OR ischemia OR occlusion) AND (intra-arterial OR endovascular OR thrombectomy OR aspiration OR stent-retriever OR reperfusion OR recanalization)”. The reference sections of prior systematic reviews and meta-analyses were also screened for related studies.

Studies were included if they met the following criteria: (1) researched a population with acute ischemic stroke (AIS) caused by BAO; (2) underwent EVT plus SMT as the intervention arm and SMT alone as the control arm; and (3) reported outcomes related to the modified Rankin scale (mRS), mortality, and symptomatic intracranial hemorrhage (sICH). Both RCTs and non-RCTs were included, and all incomplete clinical trials, review articles, and studies that did not use original data were excluded. After duplicate entries were removed, the titles and abstracts of the remaining studies were screened for eligibility, and the full texts of eligible studies were evaluated.

### 2.2. Data Extraction and Outcome Measures

Patient characteristics, intervention strategies, and outcome data were independently extracted by two reviewers (J.-H.C. and S.-H.L.). Information on the study design, study population, and inclusion and exclusion criteria was also retrieved. Any disagreement between the reviewers was resolved through a panel discussion with a third reviewer (L.C.).

The primary outcome was the incidence of a favorable functional outcome (defined as an mRS score of 0–3 at 90 days). The secondary outcomes were the incidence of functional independence (defined as an mRS score of 0–2 at 90 days), 90-day mortality rate, and sICH risk (as defined by each trial).

### 2.3. Risk-of-Bias Assessment

A risk-of-bias assessment was performed by two independent reviewers (J.-H.C. and S.-H.L.). For RCTs, version 2 of the Cochrane risk-of-bias tool for randomized trials was used, which categorizes studies as having low risk, some concerns, or high risk of bias. For non-RCTs, we used the Risk of Bias in Non-Randomized Studies—of Interventions tool, which categorizes the risk of bias as low, moderate, or serious. Publication bias was assessed by employing funnel plots for visualization; these plots are provided in the Supplemental Material. Any disagreements were resolved through a panel discussion involving all three reviewers (J.-H.C., S.-H.L., and L.C.).

### 2.4. Statistical Analysis

All statistical analyses were conducted using Review Manager 5.4 (The Cochrane Collaboration, Oxford, UK). The summary effect sizes for outcomes comparing EVT and SMT for BAO were determined as risk ratios (RRs) through the Mantel–Haenszel method with a random-effects model. In subgroup analysis, the summary effect sizes were determined as RRs through the inverse-variance method with a random-effects model. A 95% confidence interval (CI) not including 1 was considered to indicate statistical significance. Between-study heterogeneity and inconsistency were assessed using the I2 statistic, with an I² value of 0% to 25% implying low heterogeneity, 26% to 50% suggesting moderate heterogeneity, 51% to 75% indicating substantial heterogeneity, and 76% to 100% indicating high heterogeneity.

## 3. Results

### 3.1. Literature Search Results and Study Selection

**Figure 1:**
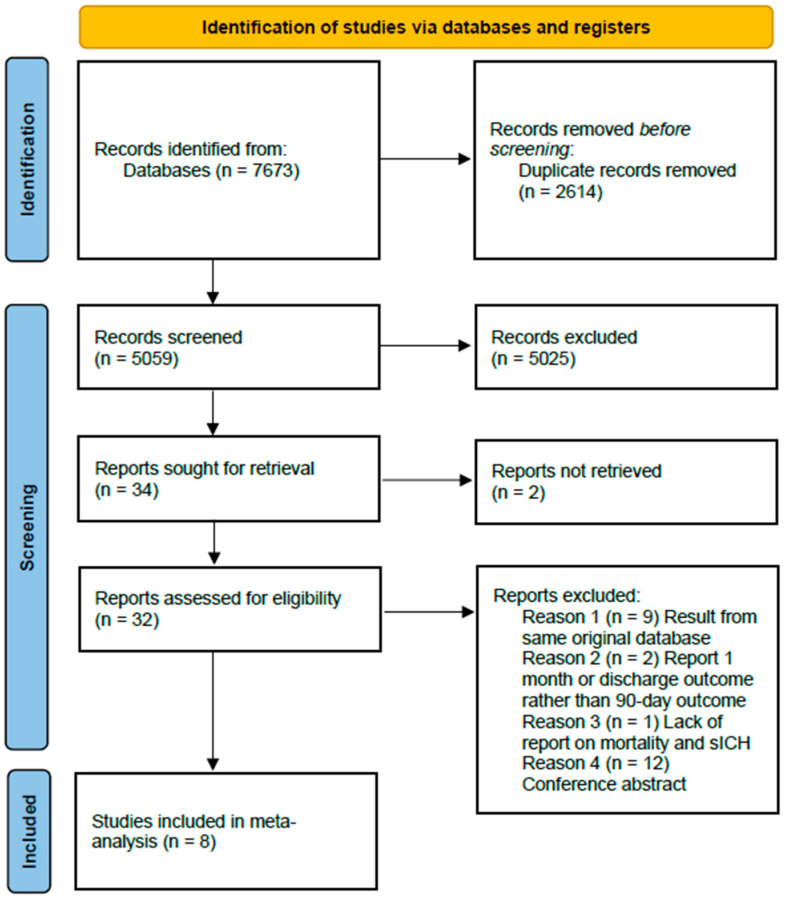
Flow chart of the study selection process.

The initial literature search yielded 7673 entries. After 2614 duplicate entries were removed, the titles and abstracts of the remaining 5059 studies were screened. Among these, 5025 studies did not meet the inclusion criteria and were excluded. Of the remaining thirty-four studies, two did not have complete texts available, and the full texts of another thirty-two were assessed for eligibility. Of them, nine were excluded because they used data from the same registry; two were removed because they used the 1-month and discharge mRS score as an outcome measurement; and one was excluded because there was no reporting data on sICH or mortality. Additionally, 12 conference abstracts were excluded. Finally, eight studies (four RCTs and four non-RCTs) were included in the meta-analysis [11,12,13,14,15,16,17,18]. The study selection flowchart is presented in Figure 1.

Table 1 presents the characteristics of the included studies. Of the four RCTs, three were conducted in China, and one was conducted internationally across seven countries. These RCTs were published between 2020 and 2022, with sample sizes ranging from 131 to 340. The median age ranged from 62 to 68 years, and the median National Institutes of Health Stroke Scale (NIHSS) score ranged from 19 to 32. The median posterior circulation Acute Stroke Prognosis Early Computed Tomography Score (pc-ASPECTS) ranged from 8 to 10. The four non-RCTs were from China, Japan, and Australia and were published between 2013 and 2022. Their sample sizes ranged from 99 to 2134, with a mean patient age of 64–77 years and a mean NIHSS score of 4–27.

### 3.2. Risk of Bias

All four RCTs were classified as having a high overall risk of bias due to deviations from the intended interventions. Among non-RCTs, one had a serious risk of bias due to confounding, all had a serious risk of bias in participant selection, and all had a serious risk of bias due to deviation from the intended interventions. Thus, all non-RCTs were also classified as having a high overall risk of bias. Funnel plots indicated no publication bias (Figures S1–S4). The details of the risk-of-bias assessment are provided in the Supplemental Material (Tables S1 and S2).

### 3.3. Effect of EVT on Functional Outcomes

**Figure 2:**
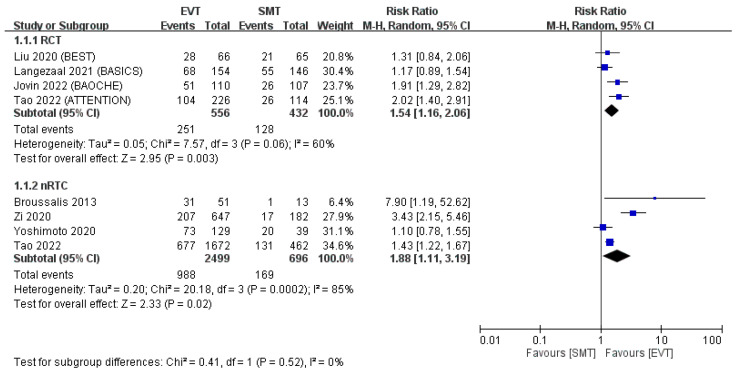
mRS score of 0–3 at 90 days. The forest plot demonstrated that the EVT group had a higher incidence of mRS scores of 0–3 at 90 days in the pooled analysis of RCTs and non-RCTs [11,12,13,14,15,16,17,18].

A pooled analysis of four RCTs including 988 patients revealed that the EVT group had a higher proportion of patients with mRS scores of 0–3 at 90 days than did the SMT group (RR: 1.54, 95% CI: 1.16–2.06; Figure 2). The average percentage of patients achieving a favorable functional outcome from EVT and SMT was 45.1% and 29.6%, respectively. Similarly, the pooled analysis of non-RCTs involving 3195 patients also revealed that EVT resulted in a higher proportion of patients with mRS scores of 0–3 at 90 days (RR: 1.88, 95% CI: 1.11–3.19; Figure 2), with the average percentage of patients achieving a favorable functional outcome being approximately 39.5% after EVT and 24.3% after SMT.

**Figure 3:**
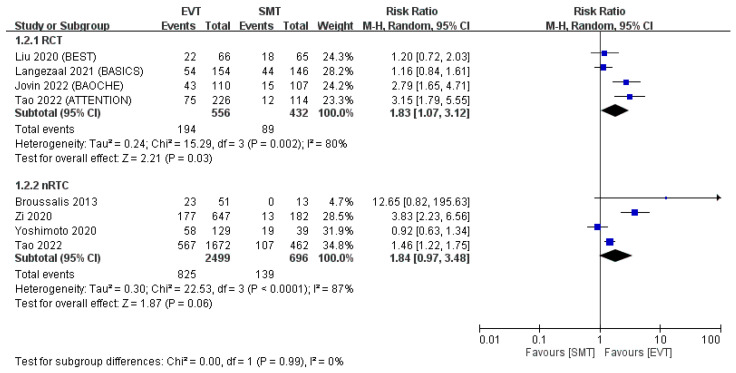
mRS score of 0–2 at 90 days. The forest plot demonstrated that the EVT group had a significantly higher incidence of mRS scores of 0–2 at 90 days in the pooled analysis of RCTs and a nonsignificantly higher incidence in the pooled analysis of non-RCTs [11,12,13,14,15,16,17,18].

A pooled analysis of RCTs indicated that the EVT group had a higher percentage of patients who achieved functional independence (mRS score of 0–2) after 90 days than did the SMT group (RR: 1.83, 95% CI: 1.07–3.12; Figure 3). However, the pooled analysis of non-RCTs indicated that the EVT group had only a nonsignificantly higher percentage of patients with functional independence at 90 days (RR: 1.84, 95% CI: 0.97–3.48; Figure 3). Overall, the average percentage of patients with mRS scores of 0–2 at 90 days was 34.9% in RCTs and 33.0% in non-RCTs in the EVT group, and only 20.6% and 20.0%, respectively, in the SMT group.

### 3.4. Effect of EVT on Mortality and sICH

**Figure 4:**
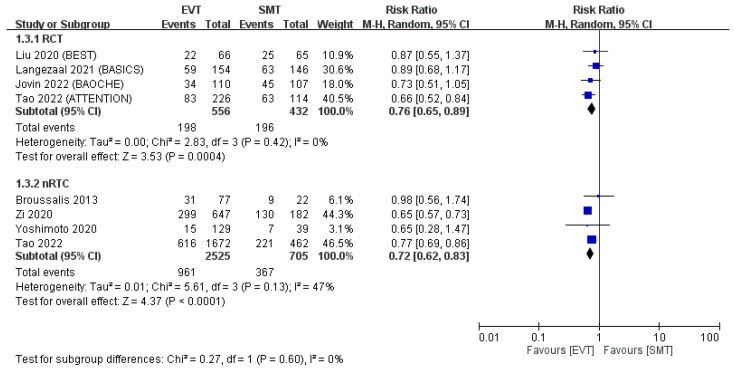
Mortality at 90 days. The 90-day mortality rate was significantly lower in the EVT group than in the SMT group in the pooled analysis of both RCTs and non-RCTs [11,12,13,14,15,16,17,18].

Pooled analysis revealed fewer 90-day mortality events in the EVT group than in the SMT group (RCTs: RR: 0.76, 95% CI: 0.65–0.89; non-RCTs: RR: 0.72, 95% CI: 0.62–0.83; Figure 4). In RCTs, the average mortality rate was 35.6% after EVT and 45.4% after SMT. In non-RCTs, these values were 38.1% and 52.1%, respectively.

**Figure 5:**
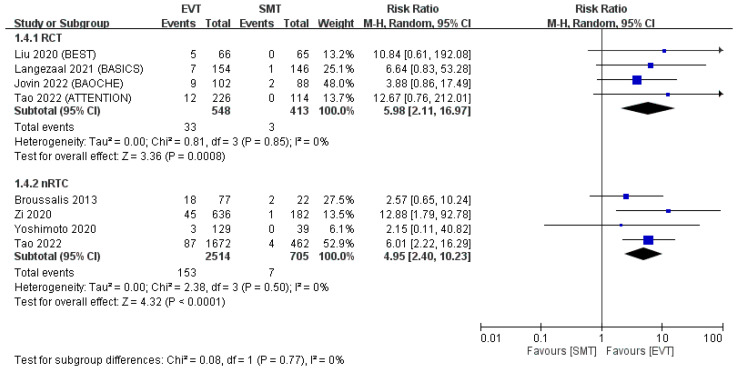
Risk of sICH. The incidence of sICH was significantly higher in the EVT group than in the SMT group in the pooled analysis of both RCTs and non-RCTs [11,12,13,14,15,16,17,18].

The EVT group had a higher incidence of sICH than did the SMT group (RCTs: RR: 5.98, 95% CI: 2.11–16.97; non-RCTs: RR: 4.95, 95% CI: 2.40–10.23; Figure 5). The average sICH risk was 6% in RCTs and 6.1% in non-RCTs in the EVT group, and only 0.7% and 1.0%, respectively, in the SMT group.

### 3.5. Factors Influencing the Functional Outcomes of EVT

Factors influencing functional outcomes were evaluated independently. The EVT group had a higher percentage of favorable functional outcome in patients aged >70 years than did the SMT group (RR: 1.75, 95% CI: 1.24–2.47; Figure S5), and also in both sexes (male: RR: 1.85, 95% CI: 1.37–2.50; female: RR: 1.93, 95% CI: 1.19–3.13; Figure S6). Although patients with an NIHSS score <10 did not benefit from EVT, the incidence of favorable functional outcomes was higher among those with an NIHSS score of ≥10 and ≥20 (RR: 1.78, 95% CI: 1.41–2.25; RR: 2.71, 95% CI: 1.44–5.09; respectively, Figure S7).

Regarding bridging therapy, intravenous thrombolysis (IVT) was associated with better functional outcomes (RR: 1.36, 95% CI: 1.05–1.76; Figure S8). Notably, a time to randomization of <6 h did not provide further benefit in the EVT group than it did in the SMT group (RR: 1.47, 95% CI: 0.90–2.39; Figure S9). The EVT group had a higher percentage of patients achieving a favorable functional outcome in the subgroups of patients with proximal and middle BAO (RR: 2.03, 95% CI: 1.42–2.91; RR: 1.50, 95% CI: 1.05–2.16; respectively, Figure S10) and with a pc-ASPECTS of ≥8 (RR: 1.46, 95% CI: 1.04–2.04; Figure S11).

## 4. Discussion

This systematic review and meta-analysis investigated the outcomes of EVT and SMT in patients with AIS and BAO. In the pooled analyses of RCTs and non-RCTs, the EVT group had a higher percentage of patients achieving a favorable functional outcome than did the SMT group. The EVT group also had a significantly higher proportion of patients achieving functional independence than did the SMT group in RCTs and was non-significantly higher in non-RCTs. Furthermore, the EVT group exhibited a lower mortality rate but a significantly higher sICH risk than did the SMT group.

### 4.1. Evidence from RCTs

The Basilar Artery Occlusion Endovascular Intervention versus Standard Medical Treatment (BEST) study was the first RCT in China to compare EVT with SMT for AIS with BAO [13]. It included patients presenting within 8 h of symptom onset, but only a small percentage of patients underwent IVT (approximately 30% in both the EVT and SMT groups). The enrolled population had high levels of stroke severity, with a mean NIHSS score of 32 and 26 in the EVT and SMT groups, respectively. The results of the intention-to-treat analysis did not indicate the benefit of EVT over SMT for BAO. However, this might be due to the higher crossover rate: 22% of patients in the SMT group ended up undergoing EVT. In per-protocol and as-treated analysis, the EVT group had a higher percentage of patients with mRS scores of 0–3 at 90 days than did the SMT group. Because the BEST trial was terminated early and only 131 patients were enrolled, the analysis was underpowered; thus, the benefits of EVT may have been masked.

The Basilar Artery International Cooperation Study (BASICS) enrolled patients with BAO who presented within 6 h of symptom onset and had NIHSS scores > 10; therefore, more participants received IVT (nearly 80%) [14]. In the BASICS, the average NIHSS score (around 22 in both groups) was lower than that in the BEST trial. The incidence of a favorable functional outcome was higher in the SMT group (up to 38%), which was higher than that in the BEST study and also higher than anticipated. This result might be due to more patients receiving IVT in the BASICS. Due to slow recruitment, the trial was later extended to patients who were aged > 85 years and had NIHSS scores < 10, yet the EVT and SMT groups included only 154 and 146 patients, respectively, leading to underpowered analyses. Although the findings did not demonstrate the superiority of EVT over SMT, the possible benefits of EVT could not be excluded because 29% of patients outside the trial had undergone EVT. The long study duration (from 2011 to 2019) was also a confounding factor because many advancements were made in the EVT device in the last decade.

Compared with the BASICS and BEST trials, the BAOCHE and ATTENTION trials demonstrated conflicting results. The BAOCHE trial enrolled patients presenting 6–24 h after symptom onset, and the ATTENTION trial enrolled those presenting <12 h after symptom onset [15,16]. In both trials, a greater proportion of patients with BAO who underwent EVT, compared with those who underwent SMT, had mRS scores of 0–3 at 90 days, despite a higher rate of sICH. The crossover rate in both trials was low, indicating reliable results without significant deviation from the intended intervention. However, 55% and 44% of the EVT group patients in the BAOCHE and ATTENTION trials, respectively, received subsequent angioplasty and stenting, suggesting higher rates of atherosclerotic events in the enrolled population; atherosclerosis is believed to be more prevalent among Asian patients [19,20].

Although these four RCTs demonstrated conflicting results, a meta-analysis of their pooled data revealed better functional outcomes in the EVT group than in the SMT group. Overall, in the EVT group, 45.1% and 34.9% achieved mRS scores of 0–3 and 0–2, respectively, after 90 days. These percentages were lower than those reported in the Highly Effective Reperfusion evaluated in Multiple Endovascular Stroke Trials (HERMES) study: 62.9% and 46%, respectively [21]. Moreover, the mortality rate in the EVT group (35.6%) was also higher than that reported in the HERMES study (15.6%). A similar result was noted for the incidence of sICH (6% vs. 4.4%). Together, the worse functional outcomes and higher mortality and risk of sICH in this meta-analysis than were reported in the HERMES study indicate the difficulty in treating AIS caused by BAO.

### 4.2. Evidence from Non-RCTs

Several observational studies of EVT for BAO had been conducted prior to these RCTs and presented conflicting results. The BASICS was one of the earliest studies comparing different strategies for treating acute symptomatic BAO [10]. In the BASICS, intra-arterial treatment (IAT) was not superior to IVT or medical therapy alone at the 1-month follow-up. Moreover, patients with mild to moderate deficits might have even been harmed by IAT. However, those who subsequently received IAT usually presented with worse conditions than those who did not. The lack of an appropriate treatment protocol for patients enrolled in the BASICS and the use of first-generation endovascular devices contributed to the limitations of this study. Therefore, no recommendations for selecting IAT or IVT for patients with BAO could be made on the basis of this study.

Unlike the BASICS, most observational registry studies evaluated 90-day functional outcomes as the primary endpoint, and this is the most common strategy adopted in research today. The BASILAR study in China enrolled 829 patients with BAO from 2014 to 2019 and concluded that patients with BAO undergoing EVT within 24 h of symptom onset had better functional outcomes [11]. This result conflicted with that reported by the BASICS, and sICH risk was lower in the BASILAR study than in the BASICS (7.1% vs. 14%). In the BASILAR study, patients who underwent EVT were younger, had higher pc-ASPECTS, and had a higher prevalence of atrial fibrillation; these factors were considered indicative of greater benefits from EVT. Moreover, most participants in the BASILAR study underwent EVT rather than SMT (647 vs. 182), implying a disparity among participating centers concerning the perceived efficacy of EVT in patients with BAO.

The benefit of EVT for BAO has also been evaluated in studies with smaller sample sizes. Broussalis et al. reported that patients with BAO undergoing EVT had better functional outcomes: the proportion of patients achieving a 90-day mRS score of 0–2 was up to 50% [17]. The EVT group also had a higher percentage of patients who achieved reperfusion and a lower proportion of patients who experienced mortality. Yoshimoto et al. concluded that EVT resulted in better functional outcomes among patients with BAO and severe neurological deficits (NIHSS score ≥ 10). [18] However, the study did not demonstrate the superiority of EVT over SMT in whole patients with BAO, particularly in those with an NIHSS score < 10.

The ATTENTION study was the largest registry study enrolling patients with BAO within an estimated 24 h; it included 462 and 1672 patients in the SMT and EVT groups, respectively [12]. In the ATTENTION study, EVT was associated with significantly better functional outcomes and survival at 90 days than was SMT. As in most studies, the incidence of sICH was higher in the EVT group (5.2%). Although the investigator was not blinded to outcome assessment, the significant associations of EVT with favorable functional outcomes and lower mortality risk supported the benefit of EVT for patients with BAO. Moreover, as in most studies, the clinical benefit of EVT was greater for those with moderate to severe symptoms.

Overall, the pooled analysis from non-RCTs demonstrated the benefit of EVT for BAO, despite a lack of consistency in each trial result. The EVT group had a higher incidence of favorable functional outcomes (mRS score of 0–3 at 90 days) than that in the SMT group. The EVT group also had a nonsignificantly higher mRS score of 0–2 at 90 days. Despite the higher rate of sICH, the 90-day mortality rate in the EVT group was significantly lower than that in the SMT group, implying that EVT might provide an overall survival benefit. These results are consistent with the findings of the meta-analysis of pooled data from RCTs, further supporting the benefits and safety of EVT for BAO.

### 4.3. Effect Modification of EVT Outcomes by Specific Factors

Several factors can influence stroke outcomes in patients with BAO, including age, sex, NIHSS score, IVT, and pc-ASPECTS. In the subgroup analysis of RCTs, age and sex were not found to contribute to worse functional outcomes in patients who underwent EVT [14,15,16]. Regardless of age (age ≥ 70 or ≤70 years) or sex (men or women), patients with BAO benefitted from receiving EVT rather than SMT. The positive outcomes associated with EVT were also observed in real-world registry studies, such as the BASILAR and ATTENTION studies, further supporting the efficacy of EVT in patients with BAO, regardless of age or sex [11,12]. Thus, neither age nor sex should be used to exclude patients from undergoing EVT.

However, the NIHSS score proved to be an important factor. The NIHSS indicates stroke severity and may impact treatment decisions and functional outcomes. Several observational studies demonstrated that patients with BAO with higher NIHSS scores derived greater benefits from EVT, especially those patients with an NIHSS score > 10 [22,23]. RCTs, such as the BASICS, BAOCHE, and ATTENTION trials, also revealed better 90-day functional outcomes for patients undergoing EVT with an NIHSS score > 10 [14,15,16]. In the BASILAR and ATTENTION studies, the benefit of EVT was observed even for patients with severe neurological deficits (NIHSS score > 20) [16,23].

Notably, patients with BAO and NIHSS scores < 10 did not experience major benefits from receiving EVT. This may be because, unlike most patients with BAO, who have severe neurological deficits, those with minor symptoms may present because of the acute deterioration of chronic BAO. In such situations, attempting recanalization of the occluded artery through EVT may not provide substantial benefits, and complications may arise, such as arterial perforation, leading to intracerebral hemorrhage. Therefore, in patients with BAO with low NIHSS scores, EVT should be considered carefully. Future studies should elaborate on this nuance.

Another factor that interacted with stroke outcomes was time from symptom onset. According to the pooled analysis of RCTs, patients who underwent EVT > 6 h after the onset of stroke symptoms experienced greater benefits than did those who underwent EVT within 6 h. This finding differs from the results of a trial focused on anterior circulation strokes [21]. An explanation for this discrepancy is that most patients with stroke presenting within 6 h of symptom onset receive IVT as their first-line treatment, which may successfully dissolve the clot and restore blood flow to the brain.

The pooled analysis of RCTs revealed that IVT was associated with better functional outcomes in the EVT group. This combination appears to have a synergistic effect, leading to improved overall outcomes for certain patients. However, studies focusing on bridging therapy for the anterior circulation stroke have not revealed significant improvements in functional outcomes, except for higher reperfusion rates [24,25]. This is attributed to the nature of the anterior circulation stroke and its response to ischemia. By contrast, a posterior circulation stroke is more resistant to ischemia because of the more extensive collateral network [26]. Consequently, successful restoration of blood flow in cases of BAO may lead to superior neurological outcomes.

Appropriate selection of patients with BAO for EVT is crucial. pc-ASPECTS is widely used to evaluate the ischemic region in the posterior circulation [27,28]. In patients with a pc-ASPECTS ≥8, EVT provided a higher incidence of favorable functional outcomes than did SMT, likely because EVT allows for greater penumbral salvage. Moreover, EVT was associated with better functional outcomes for patients with proximal and middle BAO but not those with distal BAO. The potential sparing of brainstem territory, especially pons, may have contributed to this difference in the outcome.

### 4.4. Strengths and Limitations

While numerous meta-analyses have previously explored the effectiveness of EVT versus SMT in patients with BAO following the publication of studies like BAOCHE and ATTENTION, our review distinguishes itself from prior research in several key ways [29,30,31,32]. Notably, we extend our analysis beyond RCTs to encompass non-RCTs, thereby offering a more comprehensive perspective. Additionally, our study delves into various factors that impact the outcomes of EVT, a dimension that has been less explored in previous reviews. A notable strength of our research lies in its utilization of RCTs to conduct an intention-to-treat meta-analysis, a methodology chosen to mitigate deviations from the treatment plan and reduce the potential for selection bias. Conducting separate meta-analyses for RCTs and non-RCTs enabled a direct comparison of the findings, whereas combining data yielded a large sample size, generating strong evidence to support the results. This study also has several limitations. First, the high heterogeneity among included studies, especially for functional outcome assessment, precludes drawing clear conclusions and may affect the reliability of the results. However, the consistency between the results of the pooled analyses of RCTs and non-RCTs indicates that our results are robust and reliable. Second, many of the included studies had bias due to deviations from intended interventions. Specifically, more patients with BAO underwent EVT than SMT. As a result of this bias, the treatment groups were not directly comparable. However, the use of intention-to-treat analysis helped to mitigate this bias through the analysis of patients based on their randomized treatment assignment, regardless of whether they received the assigned treatment. Third, most of the included RCTs were conducted in China, potentially limiting the generalizability of our findings. This is because the prevalence of certain conditions, such as intracranial atherosclerosis, can vary across different ethnicities and geographic regions. Finally, it is worth noting that the definition of EVT varies across studies. Some studies even include intra-arterial thrombolysis as part of their EVT criteria, as exemplified by the research conducted by Broussalis et al. However, it is important to highlight that the number of such cases is quite limited, numbering 10 in total. Consequently, their contribution to the overall analysis is likely minimal.

## 5. Conclusions

In summary, our study demonstrates the superior 90-day functional outcomes and lower mortality rates associated with EVT compared with SMT in patients with BAO. It also challenges the use of age and sex as exclusion criteria for EVT and provides guidance on which patients are more likely to benefit from EVT over SMT. Furthermore, our study highlights specific factors that can help guide treatment decisions. Patients with a high NIHSS score, those who receive IVT treatment, those with proximal or middle BA occlusion, and those with a high pc-ASPECTS are more likely to benefit from EVT over SMT. These findings have important implications for clinical practice and highlight the need for further research to validate and expand upon these results.

## Figures and Tables

**Table 1:** Characteristics of included studies.

Type	Author, Year (Trial)	Country	Study Design	Population	Characteristics			
					N	EVT	N	SMT
RCT	Liu et al., 2020 (BEST) [13]	China	Multicenter, prospective, open-label trial with blinded endpoint assessment	Age ≥ 18 years, pre-mRS score of 0–2, CTA/MRA/DSA-confirmed BAO, and onset within 8 h	66	Age: 62 (50–74) years * NIHSS score: 32 (18–38) * pc-ASPECTS: 8 (7–9) * IVT: 18 (27%) Onset-to-randomization time: 246 (139–360) min *	65	Age: 68 (57–74) years * NIHSS score: 26 (13–37) * pc-ASPECTS: 8 (7–9) * IVT: 21 (32%) Onset-to-randomization time: 278 (191–387) min *
RCT	Langezaal et al., 2021 (BASICS) [14]	International	Multicenter, open-label trial with blinded outcome assessment	Age < 85 years, NIHSS score ≥ 10, CTA/MRA-confirmed BAO, and onset within 6 h (expanded to age ≥ 85 and NIHSS score < 10 due to slow recruitment)	154	Age: 66.8 ± 13.1 years ^£^ NIHSS score: 21.9 ^£^ pc-ASPECTS: NA IVT: 121 (78.6%) Onset-to-randomization time: NA	146	Age: 67.2 ± 11.9 years ^£^ NIHSS score: 22.1 ^£^ pc-ASPECTS: NA IVT: 116 (79.5%) Onset-to-randomization time: NA
RCT	Jovin et al., 2022 (BAOCHE) [15]	China	Investigator-initiated, multicenter, open-label trial with blinded outcome evaluation	Age 18–80 years, pre-mRS score of 0–1, NIHSS score ≥ 10, BAO, onset within 6–24 h (expanded to NIHSS score ≥ 6 due to slow recruitment)	110	Age: 64.2 ± 9.6 years ^£^ NIHSS score: 20 (15–29) * pc-ASPECTS: 8 (7–10) * IVT: 15 (14%) Onset-to-randomization time: 664 (512–861) min *	107	Age: 63.7 ± 9.8 ^£^ NIHSS score: 19 (12–30) * pc-ASPECTS: 8 (7–10) * IVT: 23 (21%) Onset-to-randomization time: 662 (492–838) min *
RCT	Tao et al., 2022 (ATTENTION) [16]	China	Investigator-initiated, multicenter, prospective trial	Age ≥ 18 years, NIHSS score ≥ 10, CTA/MRA/DSA-confirmed BAO, and onset within 12 h	226	Age: 66.0 ± 11.1 years ^£^ NIHSS score: 24 (15–35) * pc-ASPECTS: 9 (8–10) * IVT: 60 (27%) Onset-to-randomization time: 306 (216–432) min *	114	Age: 67.3 ± 10.2 ^£^ NIHSS score: 24 (14–35) * pc-ASPECTS: 10 (8–10) * IVT: 35 (31%) Onset-to-randomization time: 294 (210–420) min *
non-RCT	Broussalis, 2013 [17]	Austria	Prospective, nationwide registry study	Posterior circulation stroke caused by BAO	77	Age: 68 (range 32–89) years NIHSS score: 22 (range 4–28) pc-ASPECTS: NA IVT: 30 (38.9%) Duration of treatment: 259 (range 60–1080) min	22	Age: 72 (range 31–94) years NIHSS score: 23 (range 12–28) pc-ASPECTS: NA IVT: 5/22 (22.7%) Duration of treatment: 314 (range 90–720) min
non-RCT	Zi et al., 2020 (BASILAR) [11]	China	Prospective, nationwide registry study	Age ≥ 18 years and acute symptomatic and radiologically confirmed BAO	647	Age: 64 (56–73) years ^§^ NIHSS score: 27 (17–33) ^§^ pc-ASPECTS: 8 (7–9) ^§^ IVT: 119 (18.4%) Duration of treatment: 246 (132–390) min ^§^	182	Age: 67 (59–76) years ^§^ NIHSS score: 26.5 (16–33) ^§^ pc-ASPECTS: 7 (6–8) ^§^ IVT: 47/182 (25.8%) Duration of treatment: 221.5 (116.25–407) min ^§^
non-RCT	Yoshimoto et al., 2020 (RESCUE) [18]	Japan	Prospective, multicenter registry study	MRA, CTA, or DSA-confirmed acute BAO	135	Mild group Age: 77 (67–79) ^§^ NIHSS score: 7 (5–9) ^§^ pc-ASPECTS: 8 (7–9) ^§^ Duration of treatment: 313 (155–405) min ^§^ Severe group Age: 75 (66–81) years ^§^ NIHSS score: 28 (21–33) ^§^ pc-ASPECTS: 7 (6–8) ^§^ Duration of treatment: 225 (150–455) min ^§^	42	Mild group Age: 69 (62–80) years ^§^ NIHSS score: 4 (3–5) ^§^ pc-ASPECTS: 9 (7–9) ^§^ Duration of treatment: NA Severe group Age: 80 (73–84) years ^§^ NIHSS score: 25 (19–30) ^§^ pc-ASPECTS: 6 (4–10) ^§^ Duration of treatment: NA
non-RCT	Tao et al., 2022 (ATTENTION) [12]	China	Prospective, nationwide registry study	CTA/MRA/DSA-confirmed BAO, admission within 24 h of estimated onset, and pre-mRS score of ≤ 2	1672	Age: 66 (56–73) years ^§^ NIHSS score: 20.5 (13–29) ^§^ pc-ASPECTS: NA IVT: 404 (24.2%) Duration of treatment: NA	462	Age: 64 (54–72) years ^§^ NIHSS score: 21 (14–28) ^§^ pc-ASPECTS: NA IVT: 86 (18.6%) Duration of treatment: NA

Abbreviations: RCT = randomized control trial; mRS = modified Rankin scale; CTA = computed tomography angiography; MRA = magnetic resonance angiography; DSA = digital subtraction angiography; BAO = basilar artery occlusion; NIHSS = National Institutes of Health Stroke Scale; pc-ASPECTS = posterior circulation Acute Stroke Prognosis Early Computed Tomography Score; IVT = intravenous thrombolysis.* = Data are presented as median (interquartile range); ^£^ = data are presented as mean ± standard deviation; ^§^ = data are presented as mean (interquartile range).

## Data Availability

Data are available on request.

## Supplementary Materials

The following supporting information can be downloaded at: https://www.mdpi.com/article/10.3390/jcm12206444/s1, Figure S1: Funnel plot of mRS score of 0–3 at 90 days; Figure S2: Funnel plot of a mRS score of 0–2 at 90 days; Figure S3: Funnel plot of mortality at 90 days; Figure S4: Funnel plot of risk of sICH; Figure S5: Association between age and mRS score of 0–3 at 90 days; Figure S6: Association between sex and mRS score of 0–3 at 90 days; Figure S7: Association between NIHSS and mRS score of 0–3 at 90 days; Figure S8: Association between IVT and mRS score of 0–3 at 90 days; Figure S9: Association between time to randomization and mRS score of 0–3 at 90 days; Figure S10: Association between occlusion site and mRS score of 0–3 at 90 days; Figure S11: Association between pc-ASPECTS and mRS score of 0–3 at 90 days; Table S1: Risk of bias assessment for RCTs [13,14,15,16]; Table S2: Risk of bias assessment for non-RCTs [11,12,17,18].
