# Supplementary material for: Outcomes of Endovascular Treatment versus Standard Medical Treatment for Acute Ischemic Stroke with Basilar Artery Occlusion: A Systematic Review and Meta-Analysis
Source: J Clin Med. 2023 Oct 10;12(20):6444. doi: 10.3390/jcm12206444 (PMC10607126; doi:10.3390/jcm12206444)
Supplement: Supplementary file 1 [file jcm-12-06444-s001.zip › jcm-2615979-supplementary.pdf]

Supplementary Materials

Figure S1. Funnel plot of mRS score of 0–3 at 90 days

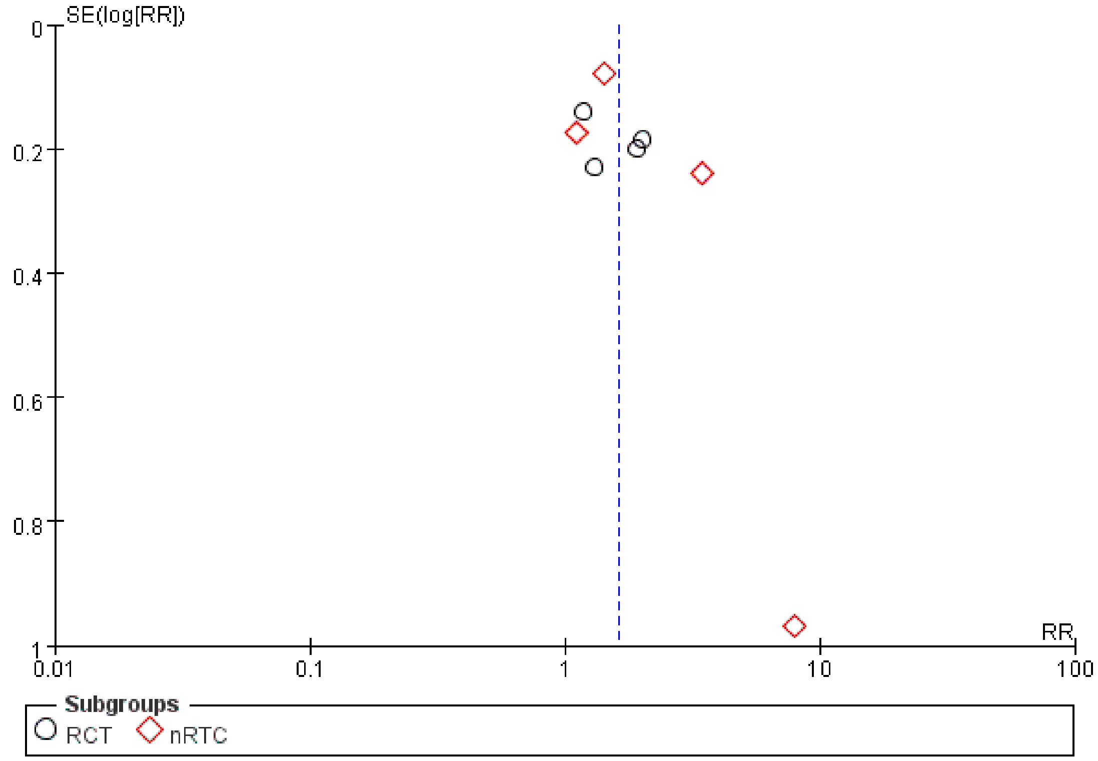

Figure S2. Funnel plot of mRS score of 0–2 at 90 days

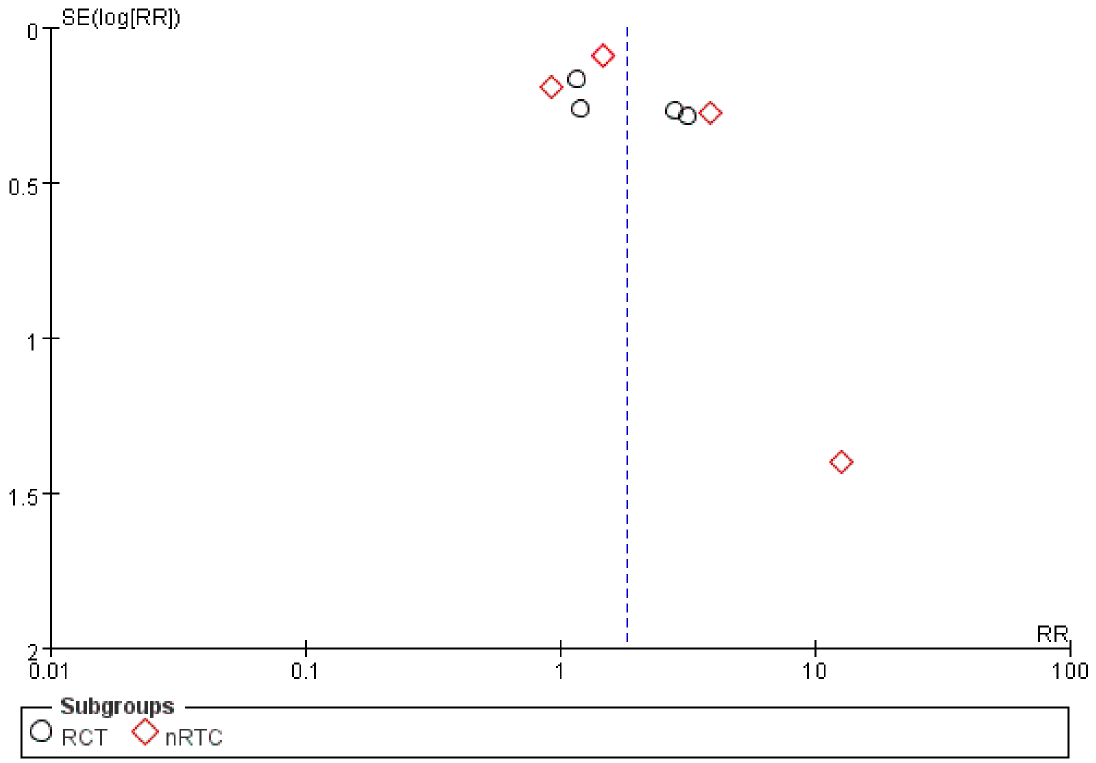

Figure S3. Funnel plot of mortality at 90 days

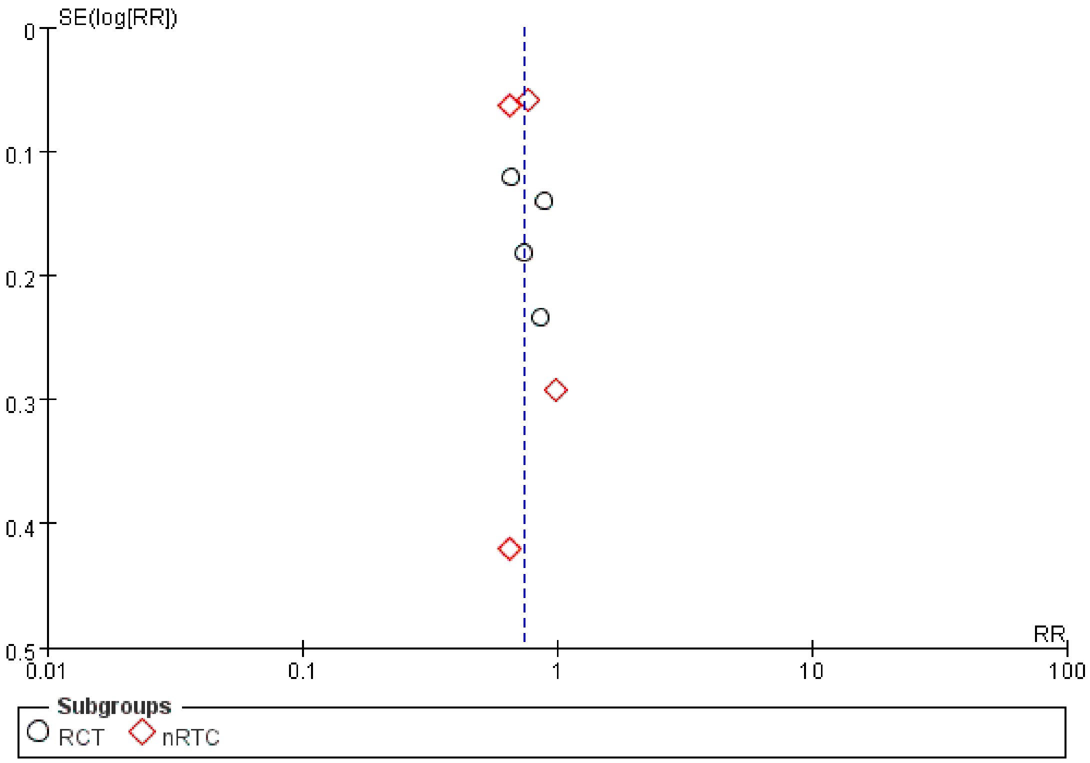

Figure S4. Funnel plot of risk of sICH

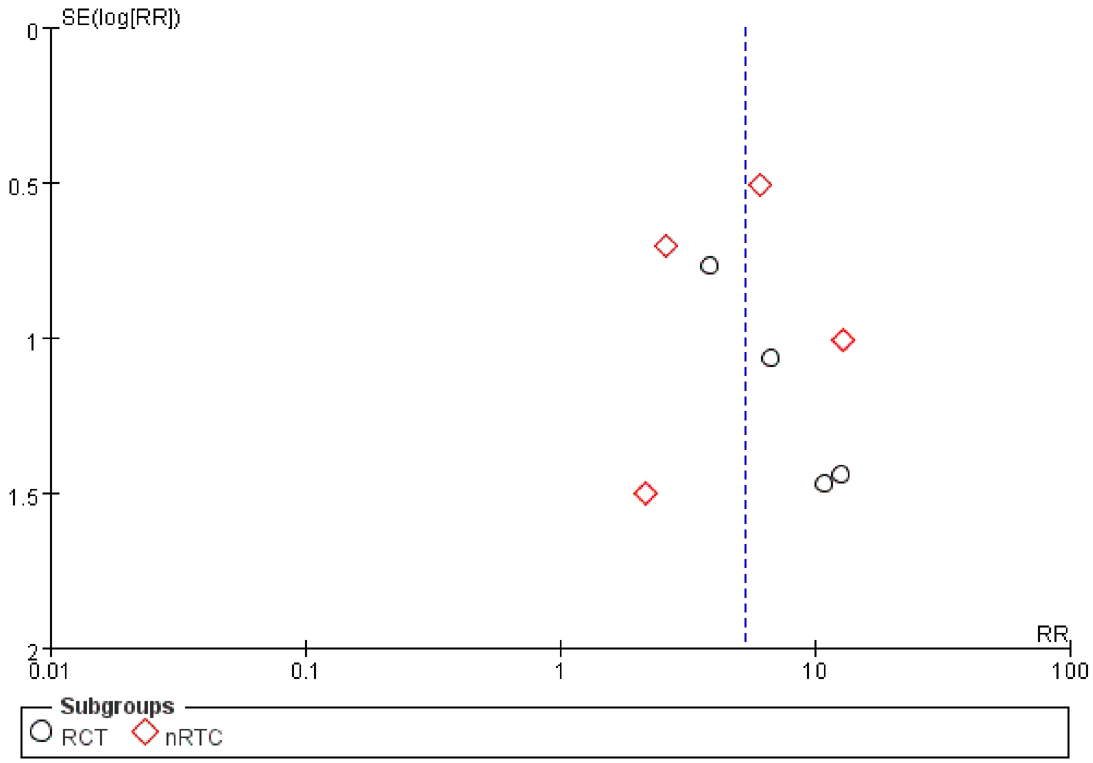

Figure S5. Interaction between age and mRS score of 0–3 at 90 days

Study or Subgroup	log[Risk Ratio]	SE	Weight	Risk Ratio IV, Random, 95% CI
**1.1.1 Age <=70**				
Langezaal 2021 (BASICS)	0.0296	0.1484	37.0%	1.03 [0.77, 1.38]
Jovin 2022 (BAOCHE)	0.5306	0.206	31.9%	1.70 [1.14, 2.55]
Tao 2022 (ATTENTION)	0.6981	0.2145	31.1%	2.01 [1.32, 3.06]
**Subtotal (95% CI)**			**100.0%**	**1.49 [0.97, 2.28]**
Heterogeneity: Tau² = 0.11; Chi² = 7.98, df = 2 (P = 0.02); I² = 75%				
Test for overall effect: Z = 1.83 (P = 0.07)				
**1.1.2 Age >= 70**				
Langezaal 2021 (BASICS)	0.3853	0.2281	59.7%	1.47 [0.94, 2.30]
Jovin 2022 (BAOCHE)	1.1119	0.6107	8.3%	3.04 [0.92, 10.06]
Tao 2022 (ATTENTION)	0.7324	0.3113	32.0%	2.08 [1.13, 3.83]
**Subtotal (95% CI)**			**100.0%**	**1.75 [1.24, 2.47]**
Heterogeneity: Tau² = 0.00; Chi² = 1.71, df = 2 (P = 0.43); I² = 0%				
Test for overall effect: Z = 3.16 (P = 0.002)				

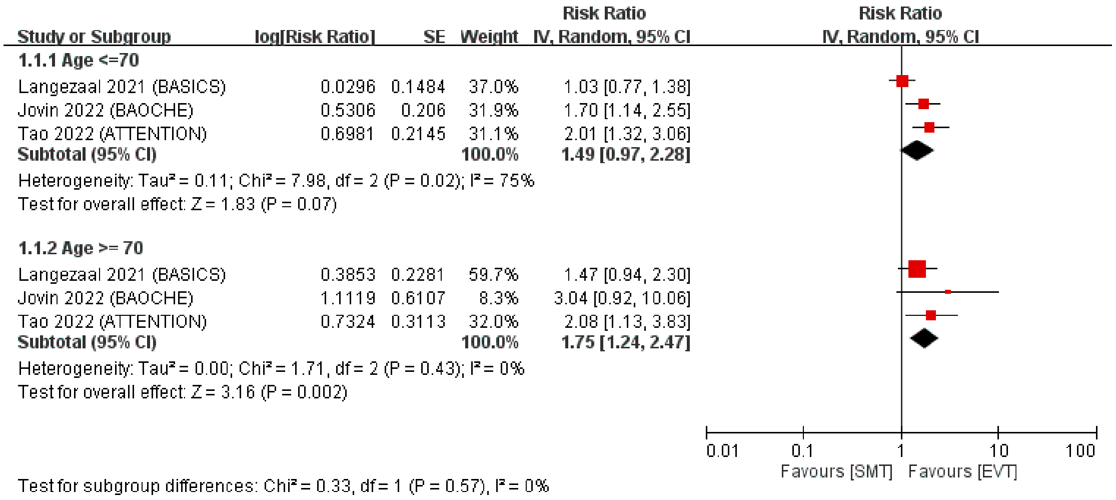

Test for subgroup differences: Chi² = 0.33, df = 1 (P = 0.57), I² = 0%

Figure S6. Interaction between sex and mRS score of 0–3 at 90 days

Study or Subgroup	log[Risk Ratio]	SE	Weight	Risk Ratio IV, Random, 95% CI
**1.2.1 Male**				
Jovin 2022 (BAOCHE)	0.4762	0.2159	50.4%	1.61 [1.05, 2.46]
Tao 2022 (ATTENTION)	0.7561	0.2178	49.6%	2.13 [1.39, 3.26]
**Subtotal (95% CI)**			**100.0%**	**1.85 [1.37, 2.50]**
Heterogeneity: Tau² = 0.00; Chi² = 0.83, df = 1 (P = 0.36); I² = 0%				
Test for overall effect: Z = 4.01 (P < 0.0001)				
**1.2.2 Female**				
Jovin 2022 (BAOCHE)	1.0818	0.5078	23.4%	2.95 [1.09, 7.98]
Tao 2022 (ATTENTION)	0.5306	0.281	76.6%	1.70 [0.98, 2.95]
**Subtotal (95% CI)**			**100.0%**	**1.93 [1.19, 3.13]**
Heterogeneity: Tau² = 0.00; Chi² = 0.90, df = 1 (P = 0.34); I² = 0%				
Test for overall effect: Z = 2.68 (P = 0.007)				

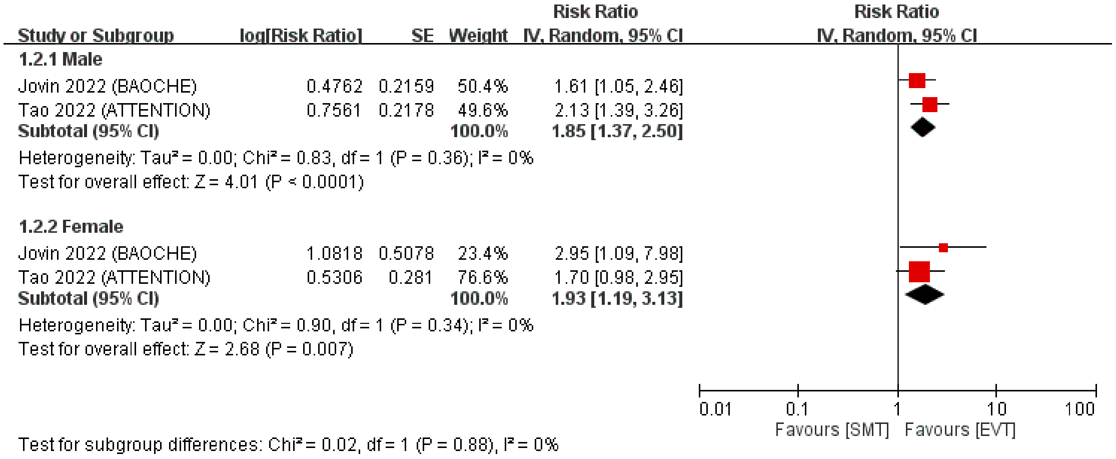

Test for subgroup differences: Chi² = 0.02, df = 1 (P = 0.88), I² = 0%

Figure S7. Interaction between NIHSS and mRS score of 0–3 at 90 days

Study or Subgroup	log[Risk Ratio]	SE	Weight	Risk Ratio IV, Random, 95% CI
**1.3.1 NIHSS < 10**				
Langezaal 2021 (BASICS)	-0.1625	0.161	55.6%	0.85 [0.62, 1.17]
Jovin 2022 (BAOCHE)	0.539	0.2855	44.4%	1.71 [0.98, 3.00]
**Subtotal (95% CI)**			**100.0%**	**1.16 [0.59, 2.30]**
Heterogeneity: Tau² = 0.19; Chi² = 4.58, df = 1 (P = 0.03); I² = 78%				
Test for overall effect: Z = 0.43 (P = 0.67)				
**1.3.2 NIHSS >= 10**				
Langezaal 2021 (BASICS)	0.3716	0.1745	39.8%	1.45 [1.03, 2.04]
Jovin 2022 (BAOCHE)	0.7309	0.2285	24.8%	2.08 [1.33, 3.25]
Tao 2022 (ATTENTION)	0.702	0.1868	35.4%	2.02 [1.40, 2.91]
**Subtotal (95% CI)**			**100.0%**	**1.78 [1.41, 2.25]**
Heterogeneity: Tau² = 0.01; Chi² = 2.29, df = 2 (P = 0.32); I² = 13%				
Test for overall effect: Z = 4.83 (P < 0.00001)				
**1.3.3 NIHSS >= 20**				
Jovin 2022 (BAOCHE)	0.6043	0.4689	40.4%	1.83 [0.73, 4.59]
Tao 2022 (ATTENTION)	1.2613	0.3698	59.6%	3.53 [1.71, 7.29]
**Subtotal (95% CI)**			**100.0%**	**2.71 [1.44, 5.09]**
Heterogeneity: Tau² = 0.04; Chi² = 1.21, df = 1 (P = 0.27); I² = 17%				
Test for overall effect: Z = 3.09 (P = 0.002)				

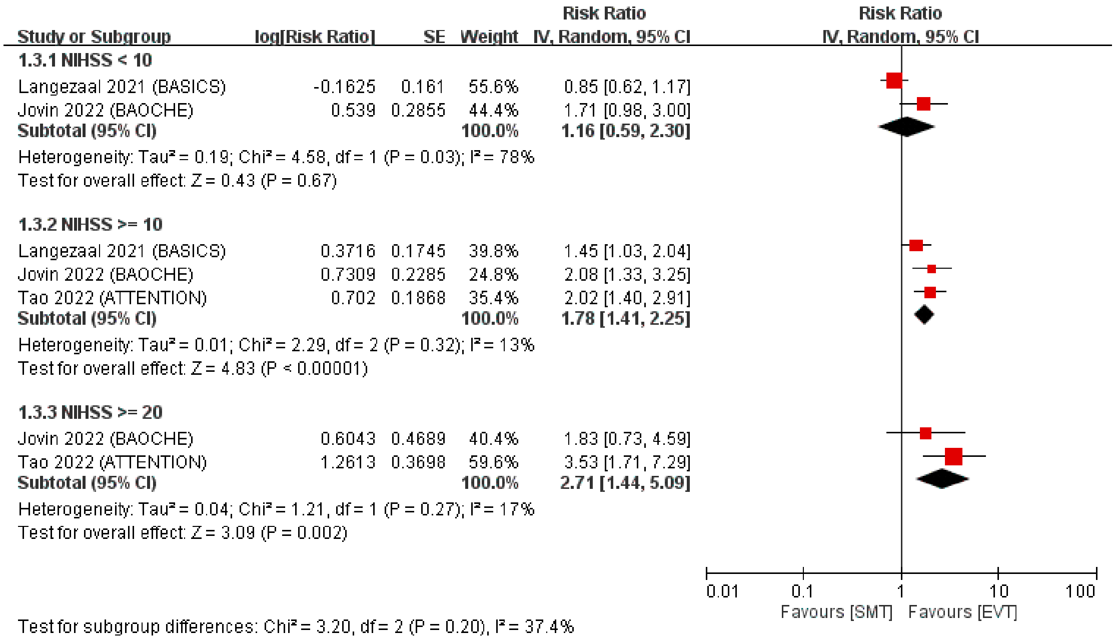

Test for subgroup differences: Chi² = 3.20, df = 2 (P = 0.20), I² = 37.4%

Figure S8. Interaction between IVT and mRS score of 0–3 at 90 days

Study or Subgroup	log[Risk Ratio]	SE	Weight	Risk Ratio IV, Random, 95% CI
**1.5.1 With IVT**				
Langezaal 2021 (BASICS)	0.2536	0.1552	71.5%	1.29 [0.95, 1.75]
Tao 2022 (ATTENTION)	0.4511	0.2457	28.5%	1.57 [0.97, 2.54]
**Subtotal (95% CI)**			**100.0%**	**1.36 [1.05, 1.76]**
Heterogeneity: Tau² = 0.00; Chi² = 0.46, df = 1 (P = 0.50); I² = 0%				
Test for overall effect: Z = 2.36 (P = 0.02)				
**1.5.2 Without IVT**				
Langezaal 2021 (BASICS)	0	0.3568	46.4%	1.00 [0.50, 2.01]
Tao 2022 (ATTENTION)	0.94	0.2527	53.6%	2.56 [1.56, 4.20]
**Subtotal (95% CI)**			**100.0%**	**1.65 [0.66, 4.15]**
Heterogeneity: Tau² = 0.35; Chi² = 4.62, df = 1 (P = 0.03); I² = 78%				
Test for overall effect: Z = 1.07 (P = 0.28)				

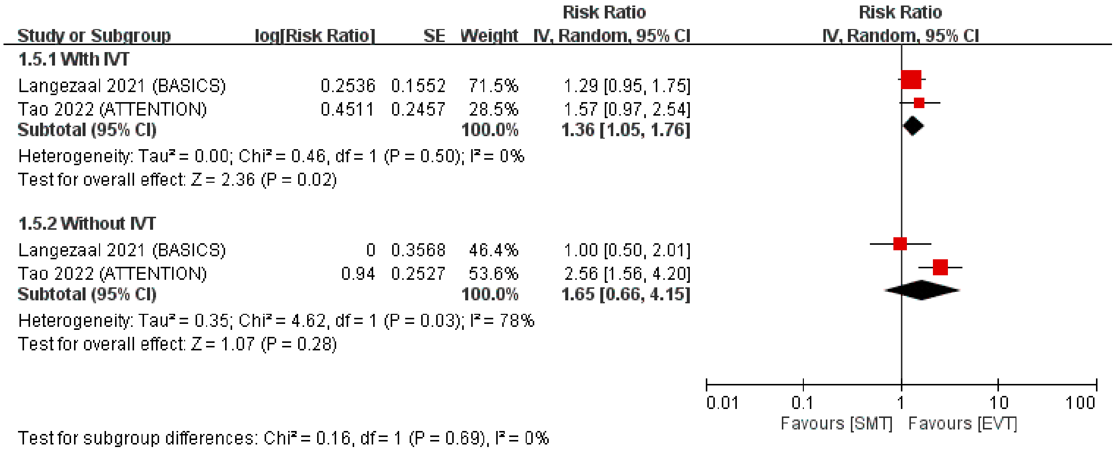

Test for subgroup differences: Chi² = 0.16, df = 1 (P = 0.69), I² = 0%

Figure S9. Interaction between time to randomization and mRS score of 0–3 at 90 days

Study or Subgroup	log[Risk Ratio]	SE	Weight	Risk Ratio IV, Random, 95% CI
**1.4.1 < 6HR**				
Langezaal 2021 (BASICS)	0.1588	0.1398	55.7%	1.17 [0.89, 1.54]
Tao 2022 (ATTENTION)	0.6627	0.2202	44.3%	1.94 [1.26, 2.99]
**Subtotal (95% CI)**			**100.0%**	**1.47 [0.90, 2.39]**
Heterogeneity: $Tau^2 = 0.09$; $Chi^2 = 3.73$, df = 1 (P = 0.05); $I^2 = 73\%$				
Test for overall effect: Z = 1.53 (P = 0.13)				
**1.4.2 > 6HR**				
Jovin 2022 (BAOCHE)	0.6461	0.1991	68.7%	1.91 [1.29, 2.82]
Tao 2022 (ATTENTION)	0.793	0.2948	31.3%	2.21 [1.24, 3.94]
**Subtotal (95% CI)**			**100.0%**	**2.00 [1.45, 2.76]**
Heterogeneity: $Tau^2 = 0.00$; $Chi^2 = 0.17$, df = 1 (P = 0.68); $I^2 = 0\%$				
Test for overall effect: Z = 4.19 (P < 0.0001)				

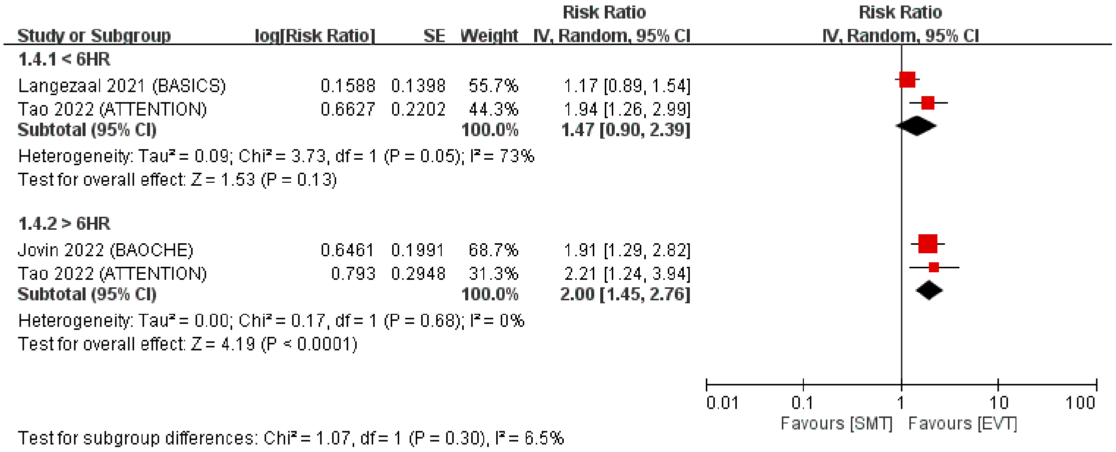

Test for subgroup differences: $Chi^2 = 1.07$, df = 1 (P = 0.30), $I^2 = 6.5\%$

Figure S10. Interaction between occlusion site and mRS score of 0–3 at 90 days

Study or Subgroup	log[Risk Ratio]	SE	Weight	Risk Ratio IV, Random, 95% CI
**1.6.1 Proximal BA**				
Langezaal 2021 (BASICS)	0.4637	0.3256	31.9%	1.59 [0.84, 3.01]
Jovin 2022 (BAOCHE)	0.6729	0.272	45.7%	1.96 [1.15, 3.34]
Tao 2022 (ATTENTION)	1.1346	0.3893	22.3%	3.11 [1.45, 6.67]
**Subtotal (95% CI)**			**100.0%**	**2.03 [1.42, 2.91]**
Heterogeneity: Tau² = 0.00; Chi² = 1.78, df = 2 (P = 0.41); I² = 0%				
Test for overall effect: Z = 3.86 (P = 0.0001)				
**1.6.2 Middle BA**				
Langezaal 2021 (BASICS)	0.2151	0.2365	56.5%	1.24 [0.78, 1.97]
Jovin 2022 (BAOCHE)	0.5128	0.3327	29.6%	1.67 [0.87, 3.21]
Tao 2022 (ATTENTION)	0.967	0.4883	14.0%	2.63 [1.01, 6.85]
**Subtotal (95% CI)**			**100.0%**	**1.50 [1.05, 2.16]**
Heterogeneity: Tau² = 0.00; Chi² = 2.07, df = 2 (P = 0.35); I² = 4%				
Test for overall effect: Z = 2.22 (P = 0.03)				
**1.6.3 Distal BA**				
Langezaal 2021 (BASICS)	-0.0202	0.1503	65.1%	0.98 [0.73, 1.32]
Jovin 2022 (BAOCHE)	-0.2768	0.5965	4.1%	0.76 [0.24, 2.44]
Tao 2022 (ATTENTION)	0.2776	0.2186	30.8%	1.32 [0.86, 2.03]
**Subtotal (95% CI)**			**100.0%**	**1.06 [0.84, 1.35]**
Heterogeneity: Tau² = 0.00; Chi² = 1.59, df = 2 (P = 0.45); I² = 0%				
Test for overall effect: Z = 0.50 (P = 0.62)				

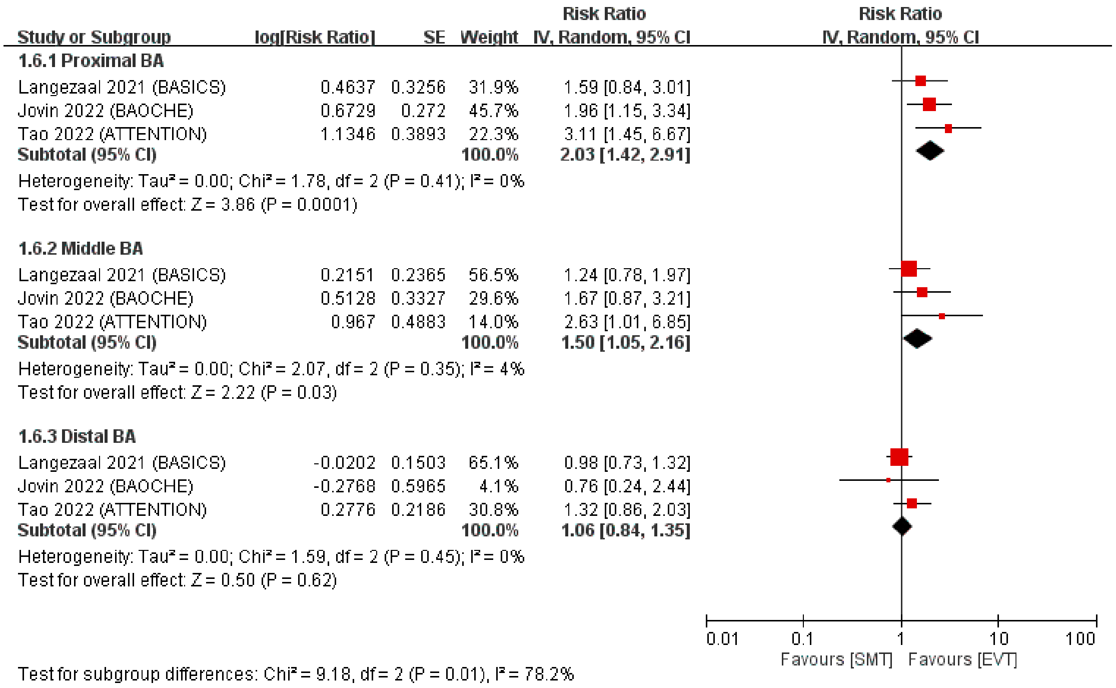

Test for subgroup differences: Chi² = 9.18, df = 2 (P = 0.01), I² = 78.2%

Figure S11. Interaction between pc-ASPECTS and mRS score of 0–3 at 90 days

Study or Subgroup	log[Risk Ratio]	SE	Weight	Risk Ratio IV, Random, 95% CI
**1.7.1 pc-ASPECTS < 8**				
Langezaal 2021 (BASICS)	0.077	0.4942	57.5%	1.08 [0.41, 2.85]
Tao 2022 (ATTENTION)	1.3507	0.6994	42.5%	3.86 [0.98, 15.20]
**Subtotal (95% CI)**			**100.0%**	**1.85 [0.54, 6.37]**
Heterogeneity: Tau² = 0.44; Chi² = 2.21, df = 1 (P = 0.14); I² = 55%				
Test for overall effect: Z = 0.98 (P = 0.33)				
**1.7.2 pc-ASPECTS >= 8**				
Langezaal 2021 (BASICS)	0.1484	0.1352	40.7%	1.16 [0.89, 1.51]
Jovin 2022 (BAOCHE)	0.3507	0.2559	24.8%	1.42 [0.86, 2.34]
Tao 2022 (ATTENTION)	0.6627	0.1775	34.5%	1.94 [1.37, 2.75]
**Subtotal (95% CI)**			**100.0%**	**1.46 [1.04, 2.04]**
Heterogeneity: Tau² = 0.06; Chi² = 5.32, df = 2 (P = 0.07); I² = 62%				
Test for overall effect: Z = 2.17 (P = 0.03)				

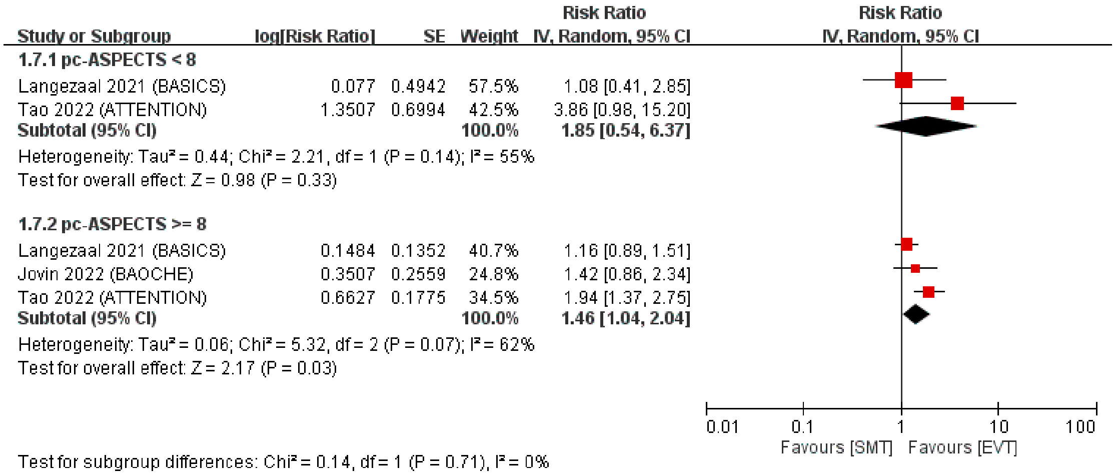

Test for subgroup differences: Chi² = 0.14, df = 1 (P = 0.71), I² = 0%

Table S1. Risk of bias assessment for RCTs

Author [Year]	Bias arising from the randomization process	Bias due to deviations from the intended interventions	Bias due to missing outcome data	Bias in measurement of the outcome	Bias in selection of the reported result	Overall risk of bias
Liu 2020 (BEST) [13]	low	high	low	low	low	high
Langezaal 2021 (BASICS) [14]	low	high	low	low	low	high
Jovin 2022 (BAOCHE) [15]	low	high	low	low	low	high
Tao 2022 (ATTENTION) [16]	low	high	low	low	low	high

Table S2. Risk of bias assessment for non-RCTs

Author [Year]	Bias due to confounding	Bias in selection of participants into the study	Bias in classification of interventions	Bias due to deviations from intended interventions	Bias due to missing data	Bias in measurement of outcomes	Bias in selection of the reported result	Overall risk of bias
Broussalis 2013 [17]	Serious	Serious	Low	Serious	Moderate	Moderate	Moderate	Serious
Zi 2020 [11]	Moderate	Serious	Low	Serious	Moderate	Moderate	Low	Serious
Yoshimoto 2020 [18]	Moderate	Serious	Low	Serious	Moderate	Moderate	Moderate	Serious
Tao 2022 [12]	Moderate	Serious	Low	Serious	Moderate	Moderate	Low	Serious
